# Forebrain neurocircuitry associated with human reflex cardiovascular control

**DOI:** 10.3389/fphys.2015.00240

**Published:** 2015-09-01

**Authors:** J. Kevin Shoemaker, Ruma Goswami

**Affiliations:** ^1^School of Kinesiology, The University of Western OntarioLondon, ON, Canada; ^2^Department of Physiology and Pharmacology, The University of Western OntarioLondon, ON, Canada

**Keywords:** cortical autonomic network, baroreflex, exercise, autonomic nervous system, medial prefrontal cortex, insula cortex

## Abstract

Physiological homeostasis depends upon adequate integration and responsiveness of sensory information with the autonomic nervous system to affect rapid and effective adjustments in end organ control. Dysregulation of the autonomic nervous system leads to cardiovascular disability with consequences as severe as sudden death. The neural pathways involved in reflexive autonomic control are dependent upon brainstem nuclei but these receive modulatory inputs from higher centers in the midbrain and cortex. Neuroimaging technologies have allowed closer study of the cortical circuitry related to autonomic cardiovascular adjustments to many stressors in awake humans and have exposed many forebrain sites that associate strongly with cardiovascular arousal during stress including the medial prefrontal cortex, insula cortex, anterior cingulate, amygdala and hippocampus. Using a comparative approach, this review will consider the cortical autonomic circuitry in rodents and primates with a major emphasis on more recent neuroimaging studies in awake humans. A challenge with neuroimaging studies is their interpretation in view of multiple sensory, perceptual, emotive and/or reflexive components of autonomic responses. This review will focus on those responses related to non-volitional baroreflex control of blood pressure and also on the coordinated responses to non-fatiguing, non-painful volitional exercise with particular emphasis on the medial prefrontal cortex and the insula cortex.

## Introduction

Moment-by-moment adjustments in cardiac function and vasomotor control are a vital component of blood pressure regulation and the distribution of blood flow. Through its sympathetic and parasympathetic branches the autonomic nervous system forms the primary neurologic effector of cardiovascular behavior. These neural mechanisms provide the basis for baseline variability in blood pressure and heart rate, or for arousal of the cardiovascular system that occurs during emotional, psychological, perceptual, or physical stressors. The pervasiveness of autonomic adjustments in human behavior, and the clinical awareness regarding the negative impact of cortical damage to cardiovascular health, have led to considerable research into the central neurocircuitry that coordinates and modulates cardiovascular control. This review deals specifically with the forebrain regions associated with cardiovascular responses to stressors involving baroreflex manipulations and volitional exercise. These conditions elicit rapid and large changes in autonomic cardiovascular adjustments. Other recent reviews can be considered for detailed information regarding the cardiodynamic behavioral outcomes that correlate with cortical activation patterns during emotional and cognitive stress (Gianaros and Sheu, 2009; Thayer and Lane, 2009; McEwen and Gianaros, 2010; Thayer et al., 2012) and the significant neuro-cardiovascular outcomes associated with cortical damage or cerebrovascular pathology (Sörös and Hachinski, 2012).

Four sections form the outline of this article. Section 1 provides an overview of the background to the developing story surrounding the cortical autonomic network. Section 2 presents a comparative neuroanatomical perspective of the insula cortex (IC) and medial prefrontal cortex (MPFC) and their role in cardiovascular arousal. Section 3 discusses the cortical autonomic network and its patterning during the physiologic maneuvers that impact cardiovascular arousal through the baroreflex or volitional exercise, models. Section 4 provides a perspective for the work on this area so far.

## Section 1: overview and clinical backdrop

Much has been determined regarding brainstem sites and pathways involved in neurocardiovascular control using electrical stimulation and tracer studies (Loewy and McKellar, 1980; Benarroch, 1993; Dampney, 1994; Potts et al., 2000; Dampney et al., 2003a). The generalized model is that afferent sensory signals synapse in the nucleus tractus solitarius, activating interneurons that disperse the signal to either the nucleus ambiguous and/or dorsal motor nucleus to affect parasympathetic outflow, or to the caudal ventrolateral medulla that inhibits the rostral ventrolateral medulla through GABAergic mechanisms. In this manner, the levels of efferent parasympathetic and sympathetic nerve traffic are manipulated. However, these brainstem pathways also receive modulatory inputs from many brainstem, subcortical and cortical centers (Barron and Chokroverty, 1993; Owens and Verberne, 2000; Castle et al., 2005). The importance of cortical modulatory influences was introduced by Claude Bernard (Cannon, 2002) who documented early anecdotal evidence from the anthropological literature that events in the human cortex can have drastic effects on the heart. He coined the term “voodoo death” to describe the higher prevalence of unexpected death in high-stress scenarios. More recently, clinical observations have advanced the concept of the “brain-heart connection.” For example, cases of sudden unexplained death in epileptic seizures (Scorza et al., 2009) and following stroke (Norris et al., 1978; Myers et al., 1982) point to neurocardiogenic dysfunction due to brain damage. Longer-term disruption of cardiovascular regulation can be observed as well, as indicated by Critchley et al. (2003) who reported reduced cardiovascular arousal in three patients following stroke isolated to the anterior cingulate cortex (ACC). In addition to acute cortical damage models, the observations of significant changes in structure, function and metabolite levels of the insula in patients with heart failure (Woo et al., 2003, 2014) and obstructive sleep apnea (Macey et al., 2008; Fatouleh et al., 2014; Yadav et al., 2014) highlight the growing understanding that cortical autonomic regions have broad-based involvement in cardiovascular disease.

In an example of reverse-translation, experimental rodent models were developed and used to explore the mechanistic details of the brain-heart connection leading to sudden death following stroke. For example, cerebrovascular occlusion in the rat middle cerebral artery led to cortical damage, particularly in the IC region, and fatal cardiac arrhythmias (Norris et al., 1978; Oppenheimer et al., 1991; Sörös and Hachinski, 2012). In their review of cortical sites affecting cardiovascular control, Cechetto and Saper (1990) provided considerable experimental evidence from rodents outlining anatomical, functional and morphological data that emphasized the role of forebrain regions, particularly the IC, in modulation of neurocardiovascular control. These observations led to a wider proposal regarding supramedullary regions involved in autonomic regulation. In 1993 Benarroch (Benarroch, 1993) proposed the “central autonomic network” which includes the IC, amygdala, hypothalamus, peri-aqueductal gray matter, parabrachial nucleus, nucleus tractus solitarius, and ventrolateral medulla, as an integrated and integral component of homeostatic regulation.

**Figure 1:**
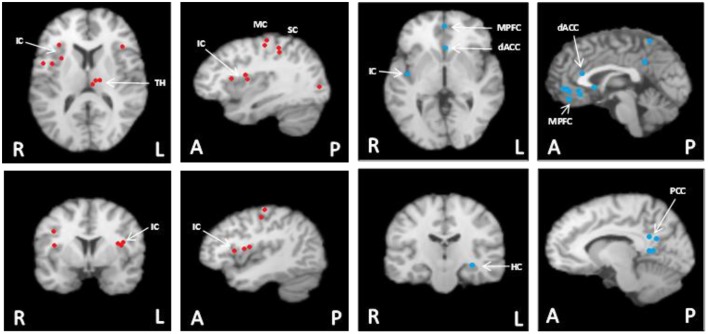
**Meta-analysis summary of data from our laboratory (n = 124) (Wong et al., 2007a,b; Al-Otaibi et al., 2010; Goswami et al., 2011; Norton et al., 2013) of common cortical regions associated with heart rate control during non-fatiguing handgrip exercise task [this figure originally published in Shoemaker et al. (2015) (used with permission)]**. These participants each performed 3–7 bouts of moderate intensity (35–40% maximal strength) handgrip tasks each lasting 30 s. Left panels with red dots: Cortical areas of increased activation relative to baseline in response to short duration, moderate intensity isometric handgrip exercise. Right panels with blue dots: Cortical areas of decreased activation relative to baseline in response to short duration, moderate intensity isometric handgrip exercise. FDR pN = 0.01; Min Volume (mm3) = 200. Analysis performed using GingerALE (Version 2.3.2; BrainMap) and Mango (Version 3.1.2; Research Imaging Institute, University of Texas Health Science Center) (Eickhoff et al., 2011; Turkeltaub et al., 2012). MPFC, medial prefrontal cortex; IC, insula cortex; dACC, dorsal anterior cingulate cortex; PCC, posterior cingulate cortex; MC, motor cortex; SC, sensory cortex; TH, thalamus; HC, hippocampus. Images are in radiological presentation with right side of the brain (R) on left, and left side of the brain (L) on the right. A, anterior; P, posterior.

The effects of general anaesthesia on autonomic function, along with limitations in the number of sites that can be examined simultaneously, represent challenges in interpreting cortical function from an experimental rodent model. Thus, an important experimental goal has been to establish the translational success of the rodent models back into the conscious human model. Two paradigms have been used to accomplish this goal. First, electrical stimulation of brain regions in awake epileptic patients with indwelling electrodes have been performed in a very limited extent and, when performed, still only expose one site at a time. Nonetheless, this model has reinforced the important role that focal regions of the IC have (or do not have) on cardiac function (Oppenheimer et al., 1992a; Al-Otaibi et al., 2010), the details of which are outlined below. More recently, the introduction of functional magnetic resonance imaging (fMRI) (Ogawa et al., 1990, 1992) has enabled studies of the temporal and spatial patterns of cortical activation patterns in conscious humans. The first fMRI report to study reflex cardiovascular control in humans (King et al., 1999) outlined a large group of forebrain regions co-activated during maximal effort maneuvers such as a Valsalva maneuver or maximal handgrip effort. Subsequently, numerous studies have begun to detail the cortical autonomic network in conscious humans, the networked nature of these sites, and their potential role in cardiovascular control. A meta-analysis of exercise-specific outcomes from our laboratory (Shoemaker et al., 2015) suggests an important role of the MPFC, IC, dorsal ACC and hippocampus (see Figure 1). However, this pattern may be specific to volitional exercise. In fact, a recent study used activation likelihood estimation meta-analysis of a number of human neuroimaging experiments and identified a set of consistently activated brain regions, comprising left amygdala, right anterior and left posterior IC and midcingulate cortices that form the core of the central autonomic network (Beissner et al., 2013). Thus, overall, a distinct group of forebrain and midbrain regions are now understood to be involved in cardiovascular control and these regions may vary from reflex to reflex.

## Section 2: comparative view

Although the cortical autonomic network includes several regions, two of these forebrain regions have received considerable attention in terms of their role in reflex cardiovascular arousal and pathology in both rodent and primate models: These include the IC and the MPFC.

### The insula cortex

#### Comparative anatomy

The IC has regions of variable cell structure or cytoarchitecture, changing from granular in the posterior portion to agranular in the mid-anterior portions (Mesulam and Mufson, 1982a,b). The anatomical and functional organization of the insula appears to follow a similar pattern. For example, horseradish peroxidase studies in the rat indicated that the anterior insula portion receives afferents from the infralimbic or medial prefrontal cortical regions, as well as the mediodorsal nucleus of the thalamus whereas ascending visceral afferents (e.g., parabrachial nuclei and parvicellular nuclei) are topographically organized in both posterior and anterior insula segments (Allen et al., 1991). The topographical organization for visceral sensory information is related to cardiovascular adjustments (Cechetto et al., 1989; Oppenheimer and Cechetto, 1990; Oppenheimer et al., 1990, 1991; Yasui et al., 1991; Butcher et al., 1993; Butcher and Cechetto, 1995; Cechetto and Chen, 1995; Cheung et al., 1997). Through these studies, and others, the reciprocal innervations of various cortical regions associated with afferent and efferent cardiovascular control were established (Cechetto and Saper, 1990; Cechetto and Shoemaker, 2009).

The primate brain exhibits similar insular anatomy. Horseradish peroxidase injected in the insula revealed labeled neurons in the prefrontal cortex, the lateral orbital region, the frontoparietal operculum, the cingulate gyrus and adjacent medial cortex, the prepiriform olfactory cortex, the temporal pole, the cortex of the superior temporal sulcus, the rhinal cortex, the supratemporal plane, and the posterior parietal lobe (Mesulam and Mufson, 1982a,b; Mufson and Mesulam, 1982). Further, tritiated amino acid injections in some of the cortical regions which contained retrograde-labeled neurons confirmed projections to the insula from prefrontal granular cortex, orbital frontal cortex, prepiriform cortex, temporal pole, rhinal cortex, cingulate gyrus, frontal operculum, and parietal cortex. Based on these observations in the monkey, Mesulam and colleagues (Mesulam and Mufson, 1982b) suggested that the posterodorsal insula was specialized for auditory-somesthetic-skeletomotor function whereas the anteroventral insula is related to olfactory-gustatory-autonomic function. In their primate measures, most of the insula, especially its anteroventral portions, had extensive interconnections with limbic structures and that through its connections with the amygdala, provides a pathway for somatosensory, auditory, gustatory, olfactory, and visceral sensations to reach the limbic system. It is conceivable that this pathway links sensory information with homeostatic autonomic reactions.

Due to methodological constraints, little direct information exists regarding the morphological connectivity of the human insula. Rather, these anatomical details are inferred largely from functional outcomes. One challenge in studying the human vs. rodent insular anatomy is the limited spatial resolution of neuroimaging techniques. Using a gyri-specific anatomical perspective, Macey et al. (2012) detailed the topographical distribution of activation patterns within the human insula during volitional tasks. The insula expresses five gyri that are organized more-or-less in a posterior-anterior direction. These five gyri responded in a temporally and regionally-specific manner to maneuvers that elicit autonomic cardiovascular arousal. For example, Valsalva stimuli appeared to be represented more anteriorly whereas volitional handgrip stimuli were associated with posterior insula activation patterns. The allocation of posterior insula to handgrip-related stimuli is also noted in other neuroimaging studies using moderate intensity handgrip (Wong et al., 2007a; Goswami et al., 2011) and with the allocation of the posterior insula to skeletomotor inputs in primates (Mesulam and Mufson, 1982a,b; Mufson and Mesulam, 1982). However, the Valsalva data, used by Macey et al. (2012) as an analog of baroreflex stress, stand in contrast to baroreflex unloading (Kimmerly et al., 2005) or loading (Cechetto and Shoemaker, 2009) stimuli which, in our experience, elicit posterior insula activation. Of note, the Valsalva maneuver requires a muscular straining effort which likely led to posterior IC activation despite fluctuations in blood pressure.

**Figure 2:**
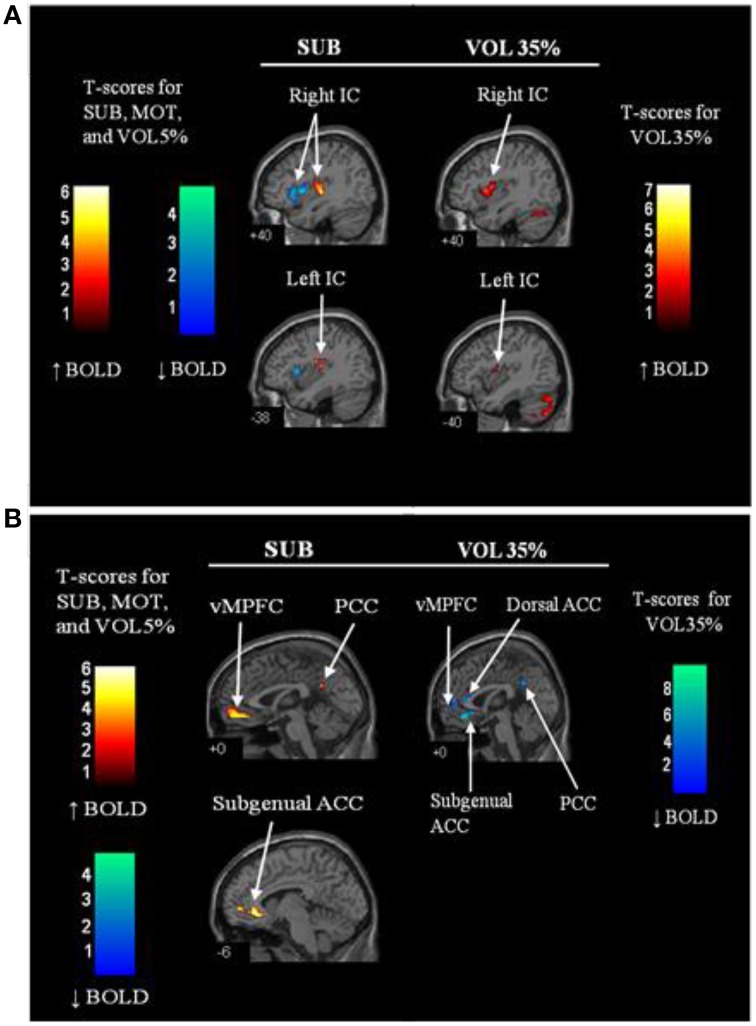
**Regional brain activation patterns in the insula (A) and midline regions (anterior cingulate (ACC) and medial prefrontal cortex (MPFC) (B) during sub-motor threshold stimulation (SUB) and 35% maximal strength volitional handgrip exercise (VOL35%) vs. rest**. Warm colors (red—orange) indicate increased activation relative to baseline whereas cool colors (blue—green) represent reduced activation relative to baseline. IC, insular cortex; vMPFC, ventral medial prefrontal cortex; ACC, anterior cingulate cortex; PCC, posterior cingulate cortex; MCC, middle cingulate cortex. *N* = 12. Adapted from Goswami et al. (2011). Used with permission.

To gain further insight into skeletomotor afferent inputs in the IC, Goswami et al. (2011) used submotor electrical stimulation of human forearm muscle during functional magnetic resonance imaging acquisition. This stimulation model is proposed to elicit activation of Type I and Type II muscle spindle afferents. When performed in supine humans, this stimulation resulted in increased activation within the posterior insula and decreased activation within the anterior insula (Goswami et al., 2011) (Figure 2). This finding corroborates that of the monkey related to skeletomotor neuroanatomy (Mufson and Mesulam, 1982) but adds the additional potential element of intra-IC communicating connections that remain poorly described. In possible contrast to the monkey, but sharing homology with the rodent, preliminary data from 10 young healthy individuals suggest that human afferent baroreceptor signals are represented within the mid-posterior superior insula (Cechetto and Shoemaker, 2009), primarily lateralized to the left insula.

Functional connectivity analysis has become a useful technique in assessing correlations between spatially segregated neural regions with activity oscillation patterns that occur at low frequencies (Fox and Raichle, 2007). Using statistical functional connectivity analysis during psychological stress imposed upon humans, Gianaros et al. (2012) reported that the left anterior insula (that region activated by the stress) exhibited functional correlations with regions implicated in autonomic function including the amygdala, pons, and the midbrain periaqueductal gray region. In addition, recent work showed disrupted resting state functional connectivity of the left IC and middle cingulate cortex after 45 days of head-down tilt bed rest (Zhou et al., 2014). These changes may disrupt autonomic balance, hemodynamic changes and/or impairments in cognition.

#### Comparative functional studies

Oppenheimer and Cechetto reported the cardiac chronotropic sites in chloralose-anesthetized rats, with tachycardia induced by stimulation of the rostral posterior IC and bradycardia in the caudal posterior IC. Both effects were abolished by atenolol but not atropine (Oppenheimer and Cechetto, 1990), and plasma catecholamines levels were modified accordingly (Oppenheimer et al., 1992b) implicating modulation of the cardiac sympathetic nervous system by these sites. The potency of this region's influence on cardiac function was demonstrated in a rat model when stimulation of the posterior insula produced atrioventricular blockade and subsequent escape rhythms, premature ventricular contractions, QT prolongation and ultimately, death in asystole (Oppenheimer et al., 1991).

The reciprocal linkages of the IC with brainstem autonomic nuclei (Verberne et al., 1997, 1988; Dampney, 1994; Owens and Verberne, 1996, 2001; Owens et al., 1999; Hasser and Moffitt, 2001; Dampney et al., 2003a,b, 2008), as observed in rodents, provide the anatomical basis for speculation that the IC may directly affect autonomic outflow. In addition, the IC may also exert its cardiovascular effects indirectly through influence of baroreflex sensitivity rather than vagal and/or sympathetic drive directly. To address this question, Zhang et al. used a damage model, with evidence that lesions of the left posterior IC in the rat increased cardiac baroreceptor gain but had no effect on baseline heart rate or blood pressure. In contrast, lesions of the right posterior IC increased baseline heart rate and blood pressure but had minimal impact on reflex gain (Zhang et al., 1998). Additional studies by this group in anesthetized monkeys outlined the presence of baroreceptor-related neurons within the IC, with greater numbers in the right than left IC (Zhang et al., 1999).

The concept of brain lateralization has gained particular interest in the context of neurocardiac function (Oppenheimer et al., 1992a, 1996) and sudden brain death (Ozdemir and Hachinski, 2008; Sörös and Hachinski, 2012). In humans, preliminary results (Cechetto and Shoemaker, 2009) indicate that the left IC may receive baroreceptor afferent input as this region displayed increased activity during transient elevations in blood pressure that followed a strong 2-s handgrip contraction (in an event-related model). These data support a role of the left posterior IC in representing baroreceptor sensory input. In contrast, the right posterior IC, together with the dorsal anterior cingulate, appears to be more involved in eliciting a baroreflex-mediated autonomic response in humans (Kimmerly et al., 2005, 2007a,b). That is, the right IC may have a diminished role in sensory integration but heightened involvement in reflex sympathetic activation.

Additional evidence regarding lateralization of IC for cardiovascular control is found in direct electrical stimulation models. Specifically, stimulation of electrodes implanted in the left IC of awake epileptic patients produced bradycardia whereas the right IC induced tachycardia (~5 beats/min) (Oppenheimer et al., 1992a, 1996). To study whether such effects could interfere with heart rate responses to stress, heart rate was measured when the left and right IC (anterior and posterior) were stimulated electrically with indwelling electrodes both at rest and during handgrip in a patient suffering from nocturnal seizures due to a benign tumor in the right posterior IC (Al-Otaibi et al., 2010). The responses were small but the pertinent outcomes indicated that the right posterior inferior IC (but not right posterior superior insula) exerted a cardio-inhibitory effect over a reflex cardiac response. These results in a young human stand in contrast to the tachycardia induced by right IC stimulation mentioned above. However, these results may point for further complexity of IC involvement in cardiovascular control in that the superior and inferior regions of the posterior IC may exert differing cardiac outcomes. Such data are difficult to obtain, focus on a singular cortical site, and are derived from the potentially damaged brain of patients. Within these limitations, the neurosurgical forebrain data support the work in animals and stroke patients pointing to an important role of the IC in cardiovascular control.

Functional magnetic resonance imaging data in conscious humans also provide support for a role of the IC in cardiac and vasomotor function. Using a model of lower body suction, Kimmerly et al. observed the cortical patterns associated with graded levels of baroreflex unloading (Kimmerly et al., 2005). Although various patterns of both increased and decreased activation were observed, baroreceptor unloading was associated with increased activation in the superior portion of the right posterior IC whereas activation within the inferior posterior region was decreased, relative to baseline, during periods when sympathetic outflow and heart rate were increased. The results from the lower body suction model corroborate the cardiac chronotropic organization of the rat (Oppenheimer and Cechetto, 1990) where regions of the posterior insula influencing tachycardia were more superior to those that elicited bradycardia. In addition, both chemical and electrical stimulation of the superior posterior IC in rodents elicited pressor effects, whereas depressor responses were observed from more caudal areas suggesting a role for this region in the regulation of efferent sympathetic cardiac function. These observations are consistent with the data presented above where electrical stimulation of the right posterior inferior IC, but not superior IC, produced bradycardia (Al-Otaibi et al., 2010). Therefore, there appears to between-species homology in terms of posterior insula function within the context of cardiovascular control.

### The medial prefrontal cortex

#### Comparative anatomy

With initial evidence from Leonard (Leonard, 1969), the proportionate homology between rats and humans has been established in terms of the anatomical region called the prefrontal cortex. Rodent tracing studies indicate that a particular region of the prefrontal cortex, called the ventral MPFC, has widespread efferent projections to brain nuclei involved in cardiovascular control. The methodologies and evidence for the connections have been reviewed comprehensively (Verberne and Owens, 1998; Resstel and Corrêa, 2006a). Briefly, the ventral MPFC sends projections that synapse with the lateral hypothalamus, cross through (no synapses) the periaqueductal gray region, converge onto the nucleus tractus solitarius and involve inhibitory synapses in the rostral ventrolateral medulla and excitatory synapses with the caudal ventrolateral medulla. Others provide evidence for the existence of a monosynaptic pathway from the vasomotor regions of the MPFC to spinal autonomic neurons (Bacon and Smith, 1993).

Very little information exists regarding the anatomical or functional linkages of the MPFC in the human. However, consistent observations have been made of reduced activation relative to baseline in the hippocampus and MFPC during volitional tasks that elicit a heart rate response. These studies have been summarized previously (Shoemaker et al., 2012a, 2015). Also, both functional (i.e., correlated in time) and effective (i.e., fluctuations in one site are correlated with, but lead, oscillations in the other) connectivity were observed in humans across a large age span (Norton et al., 2013). Beyond these studies, emphasis on the role of the hippocampus in cardiovascular dynamic responses to stress has received relatively little attention despite early studies which noted anatomical visceral sensory connections to the hippocampus (MacLean, 1952). More recently, retroviral tracing techniques established the neuronal linkages and relays that connect brainstem autonomic nuclei and the hippocampus (Westerhaus and Loewy, 2001; Castle et al., 2005). Previously, Ruit and Neafsey (1990) illustrated the ability of electrical stimulation of the ventral hippocampus to depress cardiovascular activation, exposing an inverse relationship between hippocampal activation and cardiovascular arousal. Of note, the cardiovascular outcomes in this electrical stimulation model required an intact MPFC. These findings confirm early evidence that electrical stimulation of the CA1 region of the hippocampus in anesthetized rats elicited a variety of visceral or autonomic modifications, such as decreases in heart rate and increases in pulse pressure (Kaada, 1951; Andy and Akert, 1955; Liberson and Akert, 1955; Anand and Dua, 1956). These earlier findings are supported further by recent electroencephalography results from conscious rats that point to entrainment of some theta rhythms between the hippocampus and MPFC (Hyman et al., 2011).

#### Comparative functional studies

The evidence from rodent studies points to a sympathoinhibitory default aspect of the MPFC region whereby both electrical stimulation of (Verberne, 1996; Owens et al., 1999), and acetylcholine injections into, this region evoked hypotension that was associated with sympathoinhibition and hind limb vasodilation (Crippa et al., 1999, 2000). However, the functional outcomes of stimulating this region depend upon other factors. For example, the depressor effect of ventral MPFC stimulation, outlined above, was obtained from anesthetized rodents. In contrast, both electrical (Tavares et al., 2004) and chemical stimulation with L-glutamate injections (Resstel and Corrêa, 2005) produced hypertensive and tachycardia in the anesthetized rodent. Also, removal of GABAergic inhibition of RVLM sympathoexcitatory neurons revealed a pressor response to MPFC electrical stimulation in the anesthetized rodent (Owens and Verberne, 2000). Therefore, the MPFC can distribute both pressor and depressor cardiovascular effects. Beyond these rodent studies, cholinergic modulation of cortical autonomic control in humans has not been studied in individuals with intact neural systems.

Whereas direct stimulation of the MPFC evokes acute responses, chronic lesions to the region (Verberne et al., 1987), or general inhibition by lidocaine (Resstel and Corrêa, 2006a), have no impact on baseline blood pressure or heart rate. Thus, in the rodent, it appears that this region exerts little tonic influence on cardiovascular control. This may not be the case in humans where activity in the MPFC appears to be elevated chronically. This conclusion is supported by observations of this region being active within the default mode network which represents a set of cortical regions that are active under baseline awake conditions with unfocused attention (Raichle et al., 2001). Further, volitional effort such as handgrip exercise causes a reduced activation in this region, relative to rest, in a manner that correlates highly and inversely with heart rate (Wong et al., 2007a; Goswami et al., 2011). This effect has been interpreted to reflect a disinhibition of the parasympathetic nervous system whereby chronic MPFC activation would increase parasympathetic outflow and keep baseline heart rate low (Wong et al., 2007a), or heart rate variability high (Thayer et al., 2012). In this context, removal of the MPFC brake on parasympathetic outflow would allow heart rate to increase during exercise. Evidence that NMDA receptors in the MPFC modulate parasympathetic component of the cardiac baroreflex (Resstel and Corrêa, 2006b) indicate that this forebrain region has important influence over cardiac behavior. Human studies replicate this result with evidence that activity patterns within the ventral MPFC are directly related to the high-frequency component (i.e., 0.15–0.4 Hz) component of heart rate variability (Goswami et al., 2011; Thayer et al., 2012).

The disparate findings between rodent, where MPFC has little apparent effect on baseline cardiovascular control, and human, where MPFC appears to have considerable effect on baseline heart rate, may be resolved if one considers the anesthesia effect outlined above, or the possibility that the MPFC has a role in baroreflex-mediated cardiovascular control rather than heart rate *per se*. To examine the role of the MPFC in baroreflex function investigators have measured heart rate responses to pharmacologically-induced blood pressure changes in the intact and lesioned MPFC and observed that lesions of this region impaired cardiac baroreflex sensitivity (Verberne et al., 1987; Resstel et al., 2004) by mechanisms that were localized to synapses within the MPFC (Resstel et al., 2004). Also, the MPFC region modulates baroreflex gain related to lumbar sympathetic nerve discharge in the rodent (Owens and Verberne, 2000) outlining the ability of this region to modify both cardiovagal and sympathetic arms of the baroreflex.

To summarize this section, a general pattern arises across species in terms of the insula and its morphologic and functional associations with the autonomic nervous system. Less information exists regarding the neuroanatomical linkages between MPFC and cardiovascular control centers in humans. Whereas cross-species disparity appears regarding the role of the MPFC in baseline autonomic function, the evidence suggests similar associations of this region with baroreflex function and cardiac arousal between rats and humans.

## Section 3: the cortical autonomic network and integrative reflex cardiovascular control

This section explores the collective behavior of the regions discussed above, with additional emphasis on the broader range of regional activation changes that occur during baroreceptor and exercise models of human cardiovascular arousal. There will be some redundancy in content between this section and the preceding information.

## The baroreflex

The baroreflex exerts a major influence on beat-to-beat blood pressure regulation through reflex-mediated alterations in autonomic balance that, in turn, adjust heart rate and peripheral vascular contractile state. Given the sympathetic and parasympathetic efferent arms of the reflex, the analysis of cortical circuitry associated with baroreflex function must be considered operationally within each study or methodology. It may be instructive to illustrate here that care should be taken in interpreting results of varying methods used to quantify the autonomic response. For example, efferent sympathetic outflow in humans has been quantified indirectly through blood pressure changes, changes in vascular resistance, changes in skin conductance, or more directly through microneurographic recordings of actual nerve activity. These approaches cannot be used interchangeably. Specifically, spontaneous fluctuations in sympathetic skin conductance during biofeedback maneuvers were correlated with fMRI-based assessment of activation patterns in the anterior cingulate, orbitofrontal, insular, lingual and medial prefrontal cortices, although more closely with the orbitofrontal activation (Critchley et al., 2000a; Nagai et al., 2004). However, skin conductance reflects the sympathetic changes directed to skin and is affected by emotional/psychological arousal or thermoregulatory stimuli with only weak modulation by the baroreflex. In contrast, the baroreflex exerts powerful sympathetic responses to the vasculature in skeletal muscle and the viscera, as well as chronotropic and inotropic responses in the heart. The following discussion represents situation-specific examples of cortical associations with sympathetic or cardiac control during baroreflex tests.

In humans, several procedures have been used to induce baroreflex changes during neuroimaging studies. These include: (1) volitional maneuvers such as Valsalva's maneuver, (2) direct manipulation of baroreceptor activation using either pharmacological changes in blood pressure, direct suction/pressure of the carotid sinus, or reduction in venous return through venous pooling approaches, and 3) assessment of spontaneous fluctuations in sequences heart rate and systolic blood pressure relationships that describe the “spontaneous baroreflex.”

### Valsalva's maneuver

The Valsalva maneuver reduces venous return and, therefore, stroke volume and pulse pressure. The maneuver requires the participant to produce and maintain ~30 mmHg mouth pressure for 15–20 s. Thus, this maneuver requires strong volitional effort and can be tiring in patients. Normally, four phases are used to characterize the Valsalva maneuver with each eliciting varying aspects of the baroreflex: Phase I: blood pressure rises transiently due to thoracic compression causing baroreflex activation, bradycardia, and sympathetic inhibition; Phase II: pulse pressure falls with falling venous return thereby unloading the baroreceptors leading to sympathetic activation and parasympathetic withdrawal; Phase III: release of straining maneuver eliciting an overshoot rebound rise in pressure, sympathoinhibition and bradycardia; Phase IV: the recovery and stabilization of neural and cardiovascular variables. Therefore, this maneuver elicits tremendous baroreflex stress but in short-lasting and transient patterns.

The inaugural study of King et al. (1999) illustrated the utility of fMRI to expose the complexity of cortical activation associated with large cardiovascular responses that occur during the Valsalva maneuver. In the King study, MPFC activity increased relative to a pre-test baseline period during the recovery phase (Phase III) of Valsalva's maneuver, a period of rapidly rising blood pressure and reflex-induced *declining* heart rate and sympathetic outflow. Harper et al. (2000) provided additional information, demonstrating that *increases* in heart rate during Phase II of the maneuver (when blood pressure is falling) correlated to activity in the amygdala, hippocampus, insular and lateral prefrontal cortices, dorsal pons and medulla, lentiform nucleus and the cerebellum, further implicating these sites in cardiovascular arousal with baroreflex unloading. Henderson et al. (2002) illustrated the time course of brain activation of the various regions activated during the Valsalva's maneuver further emphasizing that early recruitment occurs within limbic structures whereas slightly delayed and gradual increases were found in the dorsal brainstem, IC, and cerebellum, a time course that may mimic that in the sympathetic and cardiac chronotropic patterns. The above findings emphasize a point that such effortful maneuvers produce a distributed cortical response that shares consistency with cardiovascular patterns.

At the time of these studies, emphasis was placed on the regions that *increased* their activation levels relative to baseline; regions that *decreased* activity relative to rest were not addressed. Furthermore, the Valsalva maneuver produces marked cardiovascular changes in heart rate, blood pressure, and sympathetic outflow but these occur against a background of cognitive arousal, straining fatigue, and muscular activation. Thus, the mixture of noxious, cognitive and cardiovascular arousal will lead to a complex cortical response that will reduce specificity of the neural patterns to autonomic outcomes. Therefore, tasks that elicit more specific autonomic adjustments are needed to gain improved understanding of the cortical autonomic network in conscious humans.

### Passive baroreflex manipulation

#### Lower body suction

To examine the forebrain architecture associated with baroreflex-mediated sympathetic activation in the absence of volitional effort or changes in blood pressure, a magnetic resonance imaging compatible model of graded lower body suction was developed to simulate orthostasis in the supine participant (Kimmerly et al., 2005). With lower body suction the magnitude of venous pooling and, thereby, baroreflex unloading, can be graded so that either a change in sympathetic nerve activity occurs without a change in heart rate (e.g., < −15 mmHg suction) or both sympathetic activation and heart rate changes occur (e.g., > −30 mmHg suction). In a reproducible manner (Kimmerly et al., 2007a,b), cortical regions demonstrating increased activity that correlated with higher heart rate and greater levels of lower body suction included the right superior posterior insula, frontoparietal cortex and the left cerebellum. Conversely, using the identical statistical paradigm, bilateral anterior insular cortices, the right anterior cingulate, orbitofrontal cortex, amygdala, mediodorsal nucleus of the thalamus and midbrain showed *decreased* neural activation, relative to baseline. By separating the cortical response to lower levels of lower body suction that emphasize sympathetic activation rather than heart rate changes, these studies emphasized the strong associations between heart rate and increased activation within the right superior insula and the dorsal anterior cingulate during baroreceptor unloading. Of note, these two regions also showed a change in activation pattern following 24 h of head-down tilt bed rest in association with abnormally elevated heart rate responses to −15 mmHg lower body suction (Shoemaker et al., 2012b) highlighting the consistent observations of this insula-ACC axis in baroreflex-mediated cardiovascular control (Medford and Critchley, 2010).

#### Passive spontaneous baroreflex

The sensitivity of the baroreflex response is determined by assessing the magnitude of change in end organ response (e.g., heart rate, sympathetic nerve activity or the more indirect measure of vascular resistance or blood pressure changes), in response to a stimulus such as a change in blood pressure, carotid sinus distending pressure, atrial filling pressure, or stroke volume. Acutely, the baroreflex reflects varying degrees of sensitivity or gain depending on the levels of heart rate and the person′s arousal state or level of exertion. This baroreflex sensitivity can be quantified by various approaches (Eckberg and Fritsch, 1993) but the spontaneous sequence method (Blaber et al., 1995) is amenable to functional neuroimaging scenarios because of its higher temporal resolution that can be correlated to the blood oxygenation level dependent signal provided by fMRI. In this approach, the slope of the regression between concurrent increases or decreases in R-R interval and systolic blood pressure has been interpreted to reflect the sensitivity of the cardiovagal reflex under steady state conditions, although debate exists (Lipman et al., 2003). Mental stress evokes changes in cardiovagal baroreflex gain. Using this knowledge, Gianaros′ group (Gianaros et al., 2012) determined that, across 97 individuals, a greater suppression of baroreflex sensitivity evoked by mental stress co-varied with higher relative levels of neural activity in the dorsal ACC, posterior cingulate cortex, bilateral posterior IC, mid-insula, anterior insula, right amygdala, and right periacqueductal gray region of the mesencephalon, after controlling for age, gender, and resting baroreflex sensitivity. Overall, there appears to be consistency between the results using lower body suction and spontaneous baroreflex approaches regarding the role of the posterior superior insula and dorsal ACC in reflex cardiovascular control. However, the greater number of regions activated in the Gianaros study may reflect either the nature of the stimulus (mental stress vs. orthostatic stress) and/or the large number of participants.

## Exercise

Successful initiation and persistence of elevated physical activity requires neurally-mediated cardiovascular adjustments that elevate cardiac output, redirect blood flow, and counter the vasodilation in skeletal muscle to prevent hypotension. The role of the cortex in exercise adjustments was first suspected by Krogh and Lindhard (1913) when an anticipatory rise in both heart rate and respiration was observed prior to the exercise onset. The term “central command” was coined by these investigators who hypothesized that region(s) of the cortex provided coordinated parallel and concurrent drive to the skeletomotor, respiratory and autonomic neural systems to support the muscular activity. Pharmacological blockade studies have supported the idea that the cortex has an important role in adjusting autonomic cardiovascular variables in a manner related to the cognitive aspects of volitional work (McCloskey and Mitchell, 1972; Mitchell et al., 1989; Victor et al., 1989; Mitchell, 1990). Subsequently, neuroimaging methods have enabled more detailed studies on this hypothesis. Williamson and colleagues have used single photon emission computed tomography and functional MRI in a series of studies under conditions of volitional or imagined exercise with evidence for an involvement of the IC and anterior cingulate with the volitional aspects of exercise, independent from the blood pressure changes or muscle sensory afferents (Williamson et al., 1996, 1999, 2001, 2002, 2006).

As emphasized by Williamson′s studies, volitional exercise produces cortical activation patterns that emerge in response to the “top-down” drive to coordinate autonomic, muscle and respiratory responses as well as “bottom-up” afferent signals that are believed to coordinate an autonomic response that corrects a muscle perfusion error signal (Rowell, 1986; Rowell and O'Leary, 1990). The sensory afferents emanating from skeletal muscle do appear to exert powerful influence on autonomic adjustments that incorporate cortical autonomic pathways. Anatomically, four types of muscle sensory afferents exert autonomic influence; namely, Type I, II, III, and IV. The Type III and IV afferents are generally polymodal and unmyelinated or poorly myelinated afferents that respond to muscle tension (mostly Type III) and production of muscle metabolites or purines (mostly Type IV) (McCloskey and Mitchell, 1972; Kaufman et al., 1983; Mitchell et al., 1983). When stimulated, these afferents elicit a marked elevation in sympathetic outflow and depression in parasympathetic control over heart rate. The role of the central nervous system in mediating the cardiovascular adjustments has been established in decerebrate feline preparations (Iwamoto et al., 1985). However, the anatomical representation of these muscle afferents in the cortex, as well as the cortical circuitry related to autonomic adjustments in response to these afferents have received minimal attention, particularly in human studies. One method to isolate activation of the muscle metaboreflex afferents is to induce fatigue in the muscle and before the contraction terminates, make that muscle ischemic with a tourniquet so that blood neither flows in nor out of that muscle. In this manner, Alam and Smirk (1937) first illustrated a method to sustain muscle Type III and IV afferent activation in the absence of volitional effort. Using this model, Williamson and colleagues illustrated activation of the MPFC and IC during this post-exercise circulatory occlusion period suggesting representation of these afferents within regions of the cortical autonomic network. However, discomfort accompanies this maneuver and Macefield et al. (Macefield and Henderson, 2015) recently illustrated the point that such cortical activation patterns may represent pain processing rather than metaboreflex afferent representation *per se*. It remains possible, however, that these brain activation patterns represent an efferent autonomic response to both stimuli, or an autonomic response to the integrated stimuli.

As noted above, muscle contractions that result in Type III and IV afferent signals may also elicit a non-specific pattern of activation due to discomfort. To minimize these effects, the cortical response to brief bouts of handgrip exercise at graded moderate intensities was assessed (Wong et al., 2007a; Goswami et al., 2011). When performed by young adults at < 40% of maximal contractile force for brief periods (e.g., < 30 s) handgrip elicits an intensity-dependent tachycardia that is apparent within the first 1–2 s of the handgrip onset, growing to about 10 bpm above baseline levels over the 30 s contraction. Pharmacologic blockade evidence suggests that parasympathetic withdrawal, rather than sympathetic activation, mechanistically controls the bulk of this rapid heart rate response (Hollander and Bouman, 1975; Fagraeus and Linnarsson, 1976; Mitchell et al., 1989). It follows that regions of the brain that change their activity in association with these rapid heart rate changes may reflect sites that modulate parasympathetic chronotropic dominance. Using fMRI, the brief and modest handgrip task itself was associated with increased activation in the motor cortex, bilateral insula, thalamus, cerebellum, and basal ganglia (Wong et al., 2007a) as well as decreased activity within the posterior cingulate cortex and ventral MPFC regions. However, these early studies suggested that only the ventral MPFC correlated strongly and inversely with heart rate changes with a time course and magnitude of change that reflected variations in exercise intensities. More recently, we observed in a larger sample that both MPFC and the hippocampus are engaged during handgrip and associated with exercise-induced heart rate changes (Norton et al., 2013). Thus, the autonomic reflexive component of cortical activation during handgrip maneuvers reflect increased cortical activity in the posterior inferior bilateral insula and, more specifically, deactivation within the MPFC and hippocampus. This pattern has been observed in repeated studies (Wong et al., 2007a,b; Goswami et al., 2011) and was not affected by one's handedness or sex although females tend to produce smaller heart rate and cortical responses for the same relative workload (Wong et al., 2007a). Other reports examining the brain-heart relationship during cognitive and emotional stressors also emphasize the strong relationship between MPFC and heart responses (Critchley et al., 2000b, 2003, 2004; Critchley, 2004, 2005; Gianaros et al., 2004, 2005; Lane et al., 2009).

In addition to the ventral portion of the MPFC, previous neuroimaging studies investigating cardiovascular control reported that, in humans, tachycardia was often accompanied by increased activity at the dorsal ACC (Critchley et al., 2000a, 2003; Williamson et al., 2003; Kimmerly et al., 2005; Macefield et al., 2006). Most of these studies used maneuvers that elevated both heart rate and sympathetic outflow. As mentioned above, the use of graded lower body suction was used to isolate the sympathetic response which was linked to the posterior insula and dorsal anterior cingulate. Thus, it appears that dorsal anterior cingulate activation is a generalized response during cardiovascular arousal. Of note, the rapid heart rate response at the onset of handgrip exercise is not associated with sympathetic drive to the periphery (Mark et al., 1985; Lalande et al., 2014), although it may be present in visceral organs (Momen et al., 2003, 2005). This control feature requires additional research, because advancing age and some diseases such as heart failure, hypertension, and obesity, generally result in an unexplained elevation in sympathetic outflow (Esler, 1995; Esler et al., 1995, 2001; Zucker et al., 1995; Grassi, 1998).

Whereas Type III and IV muscle afferents activate sympathetic nervous system outflow that involve cortical autonomic regions, the Type I and II afferents from muscle spindles may exert opposing effects. These afferents are large, highly myelinated, and fast-conducting, such that they can be recruited at lower electrical thresholds than the poorly myelinated, small Type III and IV axons. Thus, as mentioned above, submotor threshold levels of muscle stimulation through anesthetized skin have been used to selectively recruit these afferents in a manner that reduced reflex-mediated sympathetic and blood pressure responses (Hollman and Morgan, 1997). The ability of this model to attenuate reflex neurovascular responses through cortical autonomic pathways was studied using fMRI (Goswami et al., 2011). As illustrated in Figure 2, during submotor stimulation of the right forearm flexors, posterior insula activity was increased suggesting a somatosensory role of this region for Type I and II afferent stimulation. Activity in the MPFC, subgenual anterior cingulate, pre and post central gyri, and supplementary motor areas, were also increased during submotor threshold stimulation. Notably, a concurrent tendency toward elevated heart rate variability was observed during submotor muscle stimulation, suggesting increased cardiovagal outflow. In contrast, MPFC activity and heart rate variability were reduced, and heart rate was increased, during volitional or effortful right handgrip exercise (35% of maximal effort). Further, differences between electrically-stimulated and volitional tasks were noted in insular and cingulate sub-regions and these were distributed along anterior–posterior and ventral–dorsal axes. The results suggest that Type I and II afferents from muscle are represented functionally in the bilateral insula, MPFC and anterior cingulate and that their functional impact is modulated by other signals during effortful contractions.

Not only are muscle afferents represented in cortical autonomic nuclei and express an ability to adjust baseline levels of cardiovagal control, they also appear to affect efferent sympathetic outflow and must interact with baroreflex pathways as well. For example, the typical responses to baroreceptor unloading through −30 mmHg lower body suction incudes elevations in both heart rate and efferent sympathetic nerve activity. These physiologic responses are associated with increased cortical activations within the mid/posterior IC, dorsal ACC, amygdala, and cerebellum, as well as decreased activity relative to rest in the MPFC/subgenual cingulate region (Goswami et al., 2012). However, when combined with submotor somatosensory afferent stimulation of the forearm flexors, the lower body suction-induced activation of the right mid/posterior IC and dorsal anterior cingulate were removed. Interestingly, the LBNP-induced activation of the posterior right insula was turned to a deactivation pattern when the suction was applied concurrently with somatosensory stimulation. Notably, this gating pattern in the cortex was coincident with a ~50% reduction in peripheral sympathetic nerve activity, illustrating a significant physiologic outcome of the gating mechanism. This action was specific to the baroreflex unloading because anterior IC and ACC activations occurring during a voluntary apnea were not modified by somatosensory stimulation (Goswami et al., 2012). Thus, these data indicate a sympathoinhibitory effect of muscle sensory stimulation during decreased baroreceptor input by a mechanism that includes conjoint insula-dorsal anterior cingulate regulation. Recall that this posterior IC-anterior cingulate pathway was linked to baroreceptor control of cardiovascular arousal in the above text. It appears that muscle somatosensory stimulation is somatotopically represented in the insula and can modulate sympathetic outflow, but only when baroreceptor inputs (also represented in the insula) are diminished. Unlike the exercise paradigm, however, somatosensory stimulation did not affect the suction-induced reduction in MPFC activity. Thus, reflex specific interactions are apparent in the manner in which ascending sensory signals modify the efferent cardiovascular control pathways and these may depend on the concurrent gating mechanisms induced by baroreceptor sensory inputs. These data further suggest existence of intra-IC connections between baroreceptor and muscle sensory receptors that target outcomes in the dorsal ACC but not the MPFC region. Further, intra-IC connections may exist between motor cortex and muscle sensory inputs that combine to modulate MPFC activity in the autonomic support of heart rate responses to volitional exercise.

## Section 4: perspectives and summary

Both basic and clinical research questions drive the need to understand the neural circuitry associated with human cardiovascular control and health. Clinical observations point to a critical involvement of the human cortex in potentially lethal cardiac arrhythmias. Furthermore, many disease states are associated with chronic depressions of cardiovagal control and chronic elevation of sympathetic outflow. These neural changes likely lead to chronic elevations of sympathetic neurotransmitters that have been linked to vascular pathology (Zukowska-Grojec et al., 1993; Zhang and Faber, 2001) and cardiac damage (Oppenheimer et al., 1990, 1991). Just how the cortical autonomic network affects such chronic changes in vagal and sympathetic outcomes is an intriguing, but unanswered, question. Rodent studies suggest that age must be considered as an important risk with evidence that advanced age enhances the impact of experimental stroke on efferent sympathetic drive and sudden cardiac death (Hachinski et al., 1992). The impact of age on the cortical autonomic network has only begun to be studied in this context in humans, but the studies based on blood oxygenation level dependent (BOLD) imaging must be considered carefully, because of the likelihood of concurrent changes in vascular and neurometabolic control.

Overall, the experimental rodent literature has outlined the important roles of several distinct cortical regions in cardiovascular control, the cellular basis of their impact, and the reciprocal anatomical linkages that explain the cardiovascular behavior associated with each site. Functional neuroimaging technologies have demonstrated in humans the breadth of cortical activity to a given stimuli and the time course of response. For the most part, the rat, monkey and human brains demonstrate considerable homology in terms of the anatomical and functional circuitry associated with cardiovascular control. Emphasized in this paper, the IC, dorsal ACC, and MPFC/subgenual anterior cingulate, are highly conserved cardiovascular control sites in the forebrain. In humans, these regions, along with the hippocampus and amygdala, associate commonly with reflex cardiovascular control (Shoemaker et al., 2015).

Ultimately, it is desirable to understand specific functions of these cortical activation patterns in terms of their inhibitory or excitatory control over the parasympathetic and sympathetic nervous system output. In some cases, such allocations appear to emerge. For example, studies that aimed to minimize changes to sympathetic drive and focus on cardiovagal control suggest that regions of the HC and MPFC exhibit chronic cardioinhibitory control, most likely through elevations in baseline parasympathetic outflow. Evidence form lower body suction studies indicate that the right posterior IC and dorsal ACC form a linkage associated with stimuli that raise sympathetic drive through reductions in baroreflex inhibition. However, many of the commonly activated regions appear to share functions or exert more integrative roles. For example, the bulk of studies indicate that the IC receives sensory information (baroreceptors, muscle sensory fibers) and “coordinates” the spread of this information to the ACC or MPFC in order to coordinate an autonomic adjustment. In this manner, the common presence of IC, ACC and MPFC regions in conditions that elicit cardiovascular arousal suggests that pathways specific to parasympathetic and sympathetic nervous system modulation are shared at the cortical level. Further, evidence provided above suggests that the associations between cortical autonomic sites and cardiovascular outcomes may act through, or be influenced by, the baroreflex pathways providing a separate level of control that integrates bottom-up and top-down inputs in the overall coordination of efferent autonomic pathways.

The overall network comprises a broader range of nuclei, including the cerebellum, thalamus, hypothalamus, basal ganglia nuclei and perhaps even motor nuclei (Bradley et al., 1991; Harper et al., 2000; Napadow et al., 2008; Barman and Gebber, 2009; Goswami et al., 2011). However, these latter regions have been related to memory, cognition and pain, but have received minimal attention in the context of neural control of human circulation. Understanding how these regions behave as a network, their sensory vs. intra-network or visceromotor roles, their inhibitory vs. excitatory function in the cortex and brainstem nuclei, their overall necessity within the context of cardiovascular control, and their plasticity in response to use patterns or risk factors, represent several unanswered questions. Important clinical questions will include those that emphasize age−, and disease-related alterations in autonomic balance that are believed to place the cardiovascular system at increased risk.

### Conflict of interest statement

The editor Vaughan G. Macefield declares that, despite currently hosting a Frontiers Research Topic with author J. Kevin Shoemaker, the review was conducted objectively. The authors declare that the research was conducted in the absence of any commercial or financial relationships that could be construed as a potential conflict of interest.
